# Prognostic values and clinical significance of POU2F3 expression in neuroendocrine carcinomas: a meta-analysis with a focus on small cell lung cancer

**DOI:** 10.1080/07853890.2025.2556253

**Published:** 2025-09-10

**Authors:** Yeoun Eun Sung, Meejeong Kim, Jihye Kim, Sung Hak Lee, Younghoon Kim

**Affiliations:** a Department of Hospital Pathology, Seoul St. Mary’s Hospital, College of Medicine, The Catholic University of Korea, Seoul, Republic of Korea; b Department of Internal Medicine and Liver Research Institute, Seoul National University College of Medicine, Seoul, Republic of Korea; c Center for Health Promotion and Optimal Aging, Seoul National University Hospital, Seoul, Republic of Korea

**Keywords:** POU2F3, small cell lung cancer, neuroendocrine carcinoma, prognosis, meta-analysis

## Abstract

**Background:**

Small cell lung cancer (SCLC) is a highly aggressive neuroendocrine carcinoma (NEC) with poor prognosis due to chemotherapy resistance. Molecular subtypes, including ASCL1, NEUROD1, YAP1 and POU2F3, have distinct clinical implications. POU2F3, linked to a tuft cell-like lineage, represents a non-neuroendocrine subtype found in SCLC and extrapulmonary NECs. However, its prognostic and clinical significance remain unclear.

**Methods:**

A meta-analysis was performed to assess the relationship between POU2F3 expression and clinical outcomes in SCLC and NECs. Studies reporting clinicopathological or survival data related to POU2F3 expression, identified *via* immunohistochemistry, were included. This review was performed following the guidelines of the Preferred Reporting Items for Systematic Reviews and Meta-Analyses (PRISMA) and was registered on the International Prospective Register of Systematic Reviews (registration number: CRD42023478789).

**Results:**

Sixteen studies involving 2011 patients met inclusion criteria. Pooled analysis revealed that POU2F3 expression in SCLC significantly improved OS (HR = 0.74, 95% CI = 0.61–0.90, *p* < .01 and PFS/RFS (HR = 0.69, 95% CI = 0.53–0.90, *p* < .01). Subgroup analyses associated POU2F3 positivity with favourable survival when surgical specimen or whole slide analysis was used, or when studies with chemotherapy regimen were included. POU2F3 expression also correlated with younger age (*p* = .02) and male sex (*p* = .03) but not with smoking history, tumour stage, nodal involvement, or vascular invasion.

**Conclusion:**

POU2F3 expression is associated with improved survival and correlates with younger age and male sex in SCLC, highlighting distinct clinical and biological characteristics of this tuft cell-originated subtype. Data on extrapulmonary NECs were limited, precluding definitive conclusions for this population.

## Introduction

Small cell lung cancer (SCLC) represents a highly aggressive neuroendocrine malignancy, accounting for a significant proportion of lung cancer diagnoses [1]. Despite initial responsiveness to platinum-based chemotherapy, SCLC is notorious for rapid acquisition of resistance and a dismal prognosis [1]. Recent advancements have highlighted the importance of molecular subtyping in SCLC, particularly with the identification of four key transcriptional subtypes: ASCL1, NEUROD1, POU2F3 and YAP1 [2]. Unlike the ASCL1- and NEUROD1-driven tumours that display classic neuroendocrine features, the POU2F3 and YAP1 subtypes are recognized as a non-neuroendocrine category [2,3]. The POU2F3 subtype, in particular, is thought to originate from a unique cellular source – a specialized tuft-cell variant – and its reliance on the POU2F3 transcription factor may confer unique therapeutic vulnerabilities [2]. However, its prognostic significance remains a subject of debate [4–8].

While the majority of neuroendocrine carcinomas (NECs) develop in the lower respiratory tract, a smaller proportion, ranging from 2% to 9%, are classified as extrapulmonary NECs [9]. Extrapulmonary NECs are known to arise from various organ systems, including the gastrointestinal, hepatobiliary, pancreatic, genitourinary and gynaecologic tracts [10,11]. Extrapulmonary NECs share similarities with their pulmonary counterparts in terms of their morphological characteristics. Their diagnosis is primarily based on the assessment of their morphological features and the utilization of immunohistochemical techniques to identify neuroendocrine markers. There have been suggestions that in extrapulmonary NEC, a POU2F3 high subtype, potentially originating from tuft cells, could be identified as a distinct classification [12].

In the landscape of SCLC and extrapulmonary NECs, the POU2F3 subtype has emerged as a distinct entity, marked by varying clinical features and prognostic outcomes. However, the prognostic impact of this subtype remains unclear due to conflicting evidence. For example, some studies have reported that POU2F3-positive tumours are associated with improved survival [5,13], whereas others found improved outcomes or no significant prognostic difference [14,15]. This disparity underscores the need for more comprehensive studies to fully elucidate the clinical implications and prognostic significance of the POU2F3 subtype. Therefore, we conducted this systematic review and meta-analysis to synthesize the current evidence and clarify the prognostic value and clinical significance of POU2F3 expression in SCLC and extrapulmonary NECs.

## Materials and methods

### Literature search strategy

Articles relevant with the subject were retrieved from electronic databases, PubMed, Embase and Web of Science until 13 June 2025. Search terms were (‘POU2F3’ or ‘SCLC-P’) and (‘carcinoma’, ‘neuroendocrine carcinoma’, ‘NEC’ or ‘neuroendocrine neoplasm’). Each article was examined carefully to determine studies with identical patient population. Investigation was conducted by two pathologists (YES and YK) and consensus was reached for any discrepancies for the cases. This review was registered on the International Prospective Register of Systematic Reviews (registration number: CRD42023478789).

### Selection criteria

The selection of studies for this meta-analysis was based on the PICOS framework as follows:

1. P (Population): The review included studies with patients diagnosed with small cell lung cancer (SCLC) or extrapulmonary neuroendocrine carcinomas (NECs).
2. I (Intervention/Exposure): The exposure of interest was the expression of POU2F3 or classification as the SCLC-P molecular subtype. For inclusion, this had to be identified using immunohistochemistry. Studies detecting POU2F3 or SCLC-P using only mRNA methods were excluded.
3. C (Comparator): The comparator groups consisted of patients with POU2F3-negative tumours or those classified under other SCLC molecular subtypes. Studies were required to present dichotomized data (POU2F3-positive vs. negative or SCLC-P vs. other subtypes) to be included.
4. O (Outcomes): The primary outcomes were survival data, specifically overall survival (OS), progression-free survival (PFS), or relapse-free survival (RFS). Studies had to report these outcomes as a hazard ratio (HR) with a 95% confidence interval (CI) or provide sufficient data to estimate them. Secondary outcomes included clinicopathological characteristics such as age, sex, smoking history, tumour histology (pure vs. combined), tumour stage, nodal involvement and vascular invasion.
5. S (Study Design): Only full-length, original research articles published in English were included. Excluded study types were abstracts, case reports, reviews, comments and animal studies.

### Data extraction and quality assessment

Two reviewers (Y.E.S. and Y.K.) independently collected data from eligible articles according to the criteria. The Newcastle–Ottawa Scale (NOS) was used to evaluate the quality of each study. Studies with a score ≥6 out of 9 were defined as qualified for further analysis. Extracted parameters were surname of first author, year of publication, primary organ, histology (SLCL, large cell lung carcinoma, or NEC), presence of combined histology, POU2F3-positivity or SCLC-P subtype, number of analysed patient samples, material used for analysis (whole slide or tissue microarray (TMA)) and clinicopathological data availability. Survival data were extracted as hazard ratio (HR) and 95% confidence intervals (CIs) from univariate Cox analysis. When only Kaplan–Meier curves were presented for survival analysis, HR and 95% CIs were estimated by using Engauge Digitizer V9.8 (http://digitizer.sourceforge.net) and spreadsheets provided by Tierney et al. [16].

### Statistical analysis

Meta-analysis was conducted using R software ver. 4.2.1 (https://cran.r-project.org/) with packages ‘meta’ and ‘dmetar’. Subgroup analysis was performed if more than two effect size (ES) shared a common subgroup. Random effect model was used for the pooled analysis while fixed effect model was used for subgroup analysis. We determined statistic heterogeneity using Cochran’s Q and *I*^2^. Sensitivity analysis was done by omitting each study to the pooled analysis. A graphical funnel plot and trim-and-fill method were used to evaluate publication bias. Statistical significance was set at *p* < .05.

## Results

### Literature search and study characteristics

**Figure 1.**
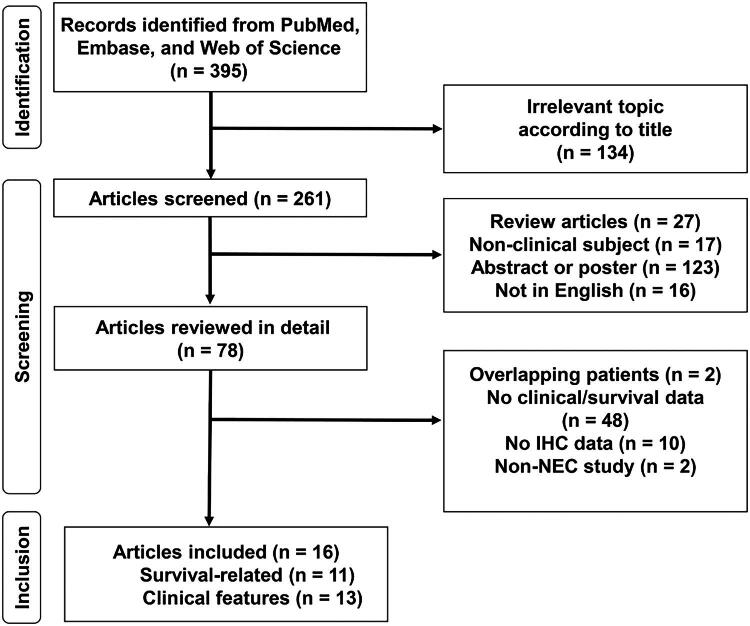
Flow chart of the selection process for meta-analysis.

Figure 1 depicts the literature search process. A total of 395 studies were searched from PubMed, Embase and Web of Science after duplicates were removed. After screening of 317 articles, 78 articles were reviewed in detail. Overall, 2011 patients within 16 studies were selected (Table 1). Among them, three studies had only survival data [13,17,18], five studies had only clinical data [19–23], and eight studies had both [4,5,8,14,15,24–26]. Two studies had two independent cohorts which were reflected as two ES in survival analysis for each study [5,13]. All survival data for the meta-analysis were either extracted from univariate Cox regression or estimated from Kaplan–Meier curve.

**Table 1.** Characteristics of articles included in the meta-analysis.

Study	Year	Availability	Survival	Clinical	Material	Total *N*	Subtype	Positivity	Organ	Histology	NOS
Ding et al. [4]	2022	Univariate cox	OS	Yes	WS	53	SCLC-P	17.0%	Lung	SCLC	7
Megyesfal et al. [5]	2022	Univariate cox	OS	Yes	WS/TMA	141/247	POU2F3	15.6%/15.1%	Lung	SCLC	6
Koh et al. [8]	2023	KM	OS	Yes	TMA	190	POU2F3	12.6%	Multiple	NEC	7
Jimbo et al. [13]	2024	KM	RFS	No	WS	95/48	SCLC-P	25.3%/29.2%	Lung	SCLC/LCC	7
Chen et al. [17]	2023	KM	PFS	No	WS	21	POU2F3	47.6%	Lung	ES-SCLC	7
Handa et al. [19]	2023	NA	NA	Yes	WS/TMA	77	SCLC-P	6.5%	Lung	SCLC	6
Matsui et al. [20]	2022	NA	NA	Yes	WS	59	SCLC-P	13.6%	Lung	SCLC	6
Shirasawa et al. [21]	2023	NA	NA	Yes	TMA	48	SCLC-P	31.3%	Lung	SCLC	6
Qi et al. [22]	2022	NA	NA	Yes	TMA	124	SCLC-P	19.4%	Lung	SCLC	6
Deng et al. [23]	2023	NA	NA	Yes	TMA	141	POU2F3	6.4%	Lung	SCLC	6
Zhu et al. [24]	2024	Univariate cox	OS/PFS	Yes	WS	195	SCLC-P	11.8%	Lung	SCLC	7
Baine et al. [14]	2022	KM	OS	Yes	WS/TMA	172	POU2F3	17.4%	Lung	SCLC	7
Li et al. [15]	2024	Univariate cox	OS/RFS	Yes	WS	58	SCLC-P	19.0%	Lung	SCLC	7
Akbulut et al. [18]	2024	KM	OS/RFS	No	TMA	116	POU2F3	20.7%	UB	NEC	7
Yu et al. [26]	2024	Univariate cox	OS	Yes	WS	117	SCLC-P	6.8%	Lung	SCLC	6
Mitsui et al. [25]	2025	Univariate cox	RFS	Yes	WS/TMA	109	SCLC-P	23.9%	Lung	SCLC/LCC	7

*Note:* NOS: Newcastle–Ottawa Scale; OS: overall survival; WS: whole slide; SLCL: small cell lung cancer; TMA: tissue microarray; KM: Kaplan–Meier curves; NEC: neuroendocrine carcinoma; RFS: regression-free survival; LCC: large cell carcinoma; PFS: progression-free survival; ES-SCLC: extensive stage small cell lung cancer; NA: not available.

### Expression of POU2F3/SLCL-P and survival

**Figure 2.**
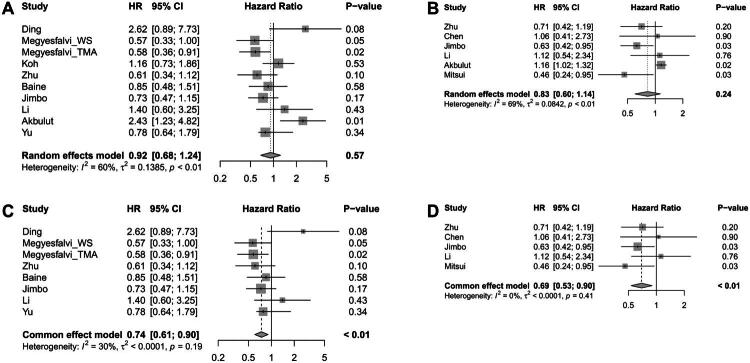
Correlation between POU2F3 expression/SCLC-P and survival in NECs. (A) Pooled analysis of overall survival (OS). (B) Pooled analysis of progression-survival (PFS)/regression-free survival (RFS). (C) Subgroup analysis for SCLC studies in OS. (D) Subgroup analysis for SCLC studies in PFS/RFS.

The meta-analysis between POU2F3 expression/SLCL-P in NECs and the prognostic value of overall survival (OS) involved nine articles with 10 ESs (Figure 2A). Pooled analysis demonstrated that having POU2F3-positivity or included as SLCL-P subtype is not associated with significantly improved survival (HR = 0.92, 95% CI = 0.68–1.24, *p* = .57). Similar to OS, pooled analysis between POU2F3/SLCL-P and progression-survival (PFS)/regression-free survival (RFS) were not significantly associated with prognosis (HR = 0.83, 95% CI = 0.60–1.14, *p* = .024) within the six available studies (Figure 2B). However, for both OS (Figure 2C) and PFS/RFS (Figure 2D), pooled analysis showed favourable prognosis for POU2F3 expression when only considering SCLC (HR = 0.74, 95% CI = 0.61–0.90, *p* < .01 and HR = 0.69, 95% CI = 0.53–0.90, *p* < .01, respectively).

Additional subgroup analysis showed that the prognostic value of POU2F3 expression could potentially vary according to specimen collection method, treatment plan, type of biomaterial and positivity definition criteria (Supplementary Figure 1). When POU2F3 expression was evaluated using only surgical specimens, POU2F3 positivity showed a statistically significant association with better OS and PFS/RFS (HR = 0.71, 95% CI = 0.56–0.90, *p* < .01 and HR = 0.58, 95% CI = 0.41–0.82, *p* < .01, respectively) (Supplementary Figure 1A) In contrast, this significant association was not observed in studies that included both surgical and biopsy specimens (HR = 1.06, 95% CI = 0.80–1.40, *p* = .70 and HR = 1.13, 95% CI = 0.99–1.28, *p* = .06, respectively) (Supplementary Figure 1B). In the subgroup of studies that included patients treated with adjuvant chemotherapy, POU2F3 expression was significantly associated with improved OS and PFS/RFS (HR = 0.79, 95% CI = 0.62–0.99, *p* = .04 and HR = 0.58, 95% CI = 0.41–0.82, *p* < .01, respectively) (Supplementary Figure 1C). However, no statistical significance was found in the subgroup treated with chemoimmunotherapy (HR = 0.91, 95% CI = 0.59–1.42, *p* = .69 and HR = 1.10, 95% CI = 0.61–1.96, *p* = .75) (Supplementary Figure 1D). In SCLC, POU2F3 expression showed a significant association with improved survival when analysed using whole slides (0.77, 95% CI = 0.61–0.98, *p* = .04 and HR = 0.74, 95% CI = 0.56–0.98, *p* = .03 for OS and PFS/RFS, respectively) or a combination of TMA and whole slides for biomaterial (0.67, 95% CI = 0.47–0.97, *p* = .03 for OS) (Supplementary Figure 1E,F). When analysing studies that defined SCLC-P (predominantly POU2F3-positive with H-score >50), pooled analysis did not show a statistically significant association with OS (HR = 0.78, 95% CI = 0.63–1.08, *p* = .17) (Supplementary Figure 1G). In contrast, when studies employing other cut-off criteria for defining POU2F3 positivity were analysed, which included H-score >10 and 1% cutoff value, POU2F3 expression demonstrated a statistically significant association with improved OS (HR = 0.64, 95% CI = 0.47–0.87, *p* < .01) (Supplementary Figure 1H). In studies that included PFS/RFS, significant correlation between extensive stage (ES)-SCLC and POU2F3 expression was not observed (1.10, 95% CI = 0.61–1.96, *p* = .75) (Supplementary Figure 1I). Subgroup analysis based on univariate Cox regression or Kaplan–Meier curve was addressed in Supplementary Figure 2.

### Expression of POU2F3/SLCL-P and clinicopathologic features

Meta-analysis for correlation between expression of POU2F3/SCLC-P and clinicopathological features of NEC is assessed in Table 2. In pooled analysis, only the sex category included a study that was not analysed from SCLC [8]. Expression of POU2F3/SCLC-P was significantly correlated with younger age and male predominance (*p* = .02 and *p* = .03, respectively). Smoking history (*p* = .56), pure versus combined histology (*p* = .87), TNM stage (*p* = .45), pN stage (*p* = .39) and vascular invasion (*p* = .98) did not show significance with POU2F3-positivity/SCLC-P.

**Table 2.** Meta-analysis between POU2F3(+)/SCLC-P and clinicopathological features.

Parameters	Studies	M-H OR	95% CI	*p* Value
Age	6	2.02	1.31–3.13	.02*
Sex (male/female)	13	1.45	1.04–2.01	.03*
Sex (SLCL only)	12	1.65	1.16–2.36	<.01*
Smoking history	9	1.30	0.52–3.29	.56
Histology (pure vs combined)	6	0.89	0.28–2.72	.87
TNM stage	10	1.15	0.78–1.66	.45
pN stage	5	1.53	0.58–4.02	.39
Vascular invasion	4	0.49	0.49–2.02	.98

*Note:* M-H OR: Mantel-Haenszel odds ratio; CI: confidence interval; TNM: tumour-node-metastasis.

* *p* < .05.

### Publication bias and sensitivity analysis

**Figure 3.**
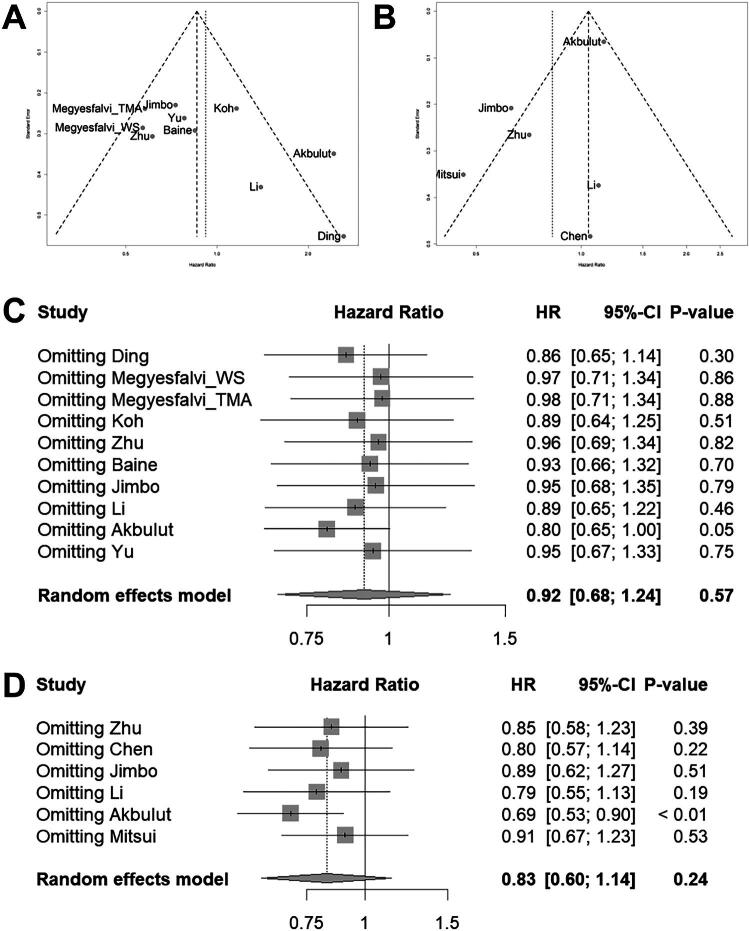
Funnel plot and sensitivity analysis. (A) Funnel plot for OS. (B) Funnel plot for PFS/RFS. (C) Sensitivity analysis for OS. (D) Sensitivity analysis for PFS/RFS.

Pooled analysis between expression of POU2F3/SLCL-P and OS or PFS/RFS showed potential asymmetry in the funnel plot, but according to Egger’s test, a significant publication bias was not suspected (*p* = .062 and *p* = .120, respectively) (Figure 3A,B).

For OS, the trim-and-fill method imputed one potentially missing study. The adjusted pooled hazard ratio (HR) using the random-effects model was 0.86 (95% CI: 0.63–1.18), which remained statistically non-significant (*p* = .36), consistent with the original analysis.

For PFS/RFS, the trim-and-fill method identified potential funnel plot asymmetry and imputed three potentially missing studies (*k* = 9). The adjusted pooled HR using the random-effects model changed from 0.83 (95% CI: 0.60–1.14, *p* = .24) to 1.15 (95% CI: 0.78–1.69, *p* = .49). While both analyses remained statistically non-significant, a reversal in the direction as well as increase in heterogeneity was detected (*I*^2^ = 68.8% to *I* ^2^ = 78.8%). Thus, interpretations regarding PFS/RFS should be more cautious.

Omitting individual ES in OS and PFS/RFS has demonstrated that robustness of the analysis is greatly affected by urinary bladder NEC study by Akbulut for both OS and PFS/RFS (Figure 3C,D) [18].

## Discussion

This systematic review and meta-analysis of 16 studies involving 2011 patients provides important insights into the prognostic significance of POU2F3 expression in SCLC and extrapulmonary NECs. Our pooled analysis revealed that POU2F3 expression is not significantly associated with OS or PFS/RFS cross all NEC, but when restricted to SCLC, POU2F3 positivity demonstrated favourable association with both OS and PFS. Subgroup analyses further indicated that the prognostic value of POU2F3 may be influenced by specimen collection methods, treatment modalities and analytical approaches, with stronger associations with prognosis observed in studies using surgical specimens and chemotherapy regimen. Additionally, POU2F3 expression was significantly associated with younger patient age and male sex, but showed no correlation with smoking history, tumour stage, nodal involvement, or vascular invasion.

The prognosis of the POU2F3 subtype remains a subject of conflicting evidence. Ding et al. reported that the SCLC-P subtype was identified as an independent poor prognostic factor in patients with resectable small cell lung cancer in a multivariate analysis. In their study, which included 53 patient samples, 9 cases (17.0%) were classified as the POU2F3 subtype [4]. Similarly, Deng et al. reported that the POU2F3 subtype was associated with an unfavourable prognosis in univariate analysis (*p* = .038). However, when adjusted for tumour stage in a multivariate Cox analysis, the association was not statistically significant (*p* = .098) [23].

Conversely, other studies reported improved or neutral prognostic implications of POU2F3 expression. In a multicentre international study by Megyesfalvi et al. patients with high POU2F3-expressing tumours exhibited significantly improved overall survival (OS) in univariate analysis (*p* = .046). In this study, the POU2F3 subtype was present in 16% of cases; however, the findings did not achieve statistical significance in multivariate analysis (*p* = .08) [5]. Similarly, Jimbo et al. observed that NECs with POU2F3 expression (26% of cases) demonstrated significantly improved relapse-free survival compared to their non-POU2F3 counterparts (*p* = .026). However, no statistically significant difference in overall survival was found (*p* = .172) [13]. Zhu et al. found no significant relationship between POU2F3 expression and prognosis, although a non-significant trend toward better outcomes was noted [24].

The conflicting evidence regarding the prognostic role of POU2F3 may be partially explained by the methodological heterogeneity between studies, which our meta-analysis sought to clarify. For instance, we found a significant association between POU2F3 expression and improved survival only in studies that used surgical specimens, whereas no significance was found when both surgical and biopsy specimens were included, as well as in ES-SCLC, in which surgical resection is not considered a primary treatment option. The lack of significance in ES-SCLC is likely attributable not only to the reliance on small biopsy specimens but also to treatment-induced temporal heterogeneity, which can alter molecular subtypes through chemotherapy or disease progression [27,28]. This suggests that the limited tissue volume in biopsies may be insufficient for accurate subtyping, potentially diluting the prognostic association. POU2F3 is known for its considerable intratumoural heterogeneity in SCLC which could affect difference in prognostic association between surgical and biopsy specimen [29]. Although studies directly comparing biopsy and surgical resection specimens are lacking, one study by Handa et al. provides valuable insight [19]. This study shows a significant correlation for SCLC subtyping among paired whole slides, single TMA cores and lymph node metastases (*p* < .001), suggesting that biopsy specimens can be useful for detecting molecular subtypes if properly sampled; for example, avoiding necrosis [19]. In addition, our subgroup analysis revealed a strong correlation between the POU2F3-positive subtype and OS, and this association was consistent whether the immunohistochemistry was based on whole slides or TMA and whole slides. However, the finding related to TMAs should be interpreted with caution, as it is based on only two studies.

Furthermore, the choice of analytical method and positivity criteria appears to influence prognostic significance. Our analysis showed that POU2F3 positivity was linked to better overall survival when specific cut-off criteria were used (H-score >10 or 1% cutoff), but not when defined by the broader SCLC-P classification with predominant POU2F3 expression and H-score >50.

POU2F3-driven small cell carcinomas are biologically distinct, characterized by the absence of classic neuroendocrine lineage markers, including synaptophysin, chromogranin and INSM1 and the expression of tuft cell lineage markers [18,30]. This unique biology suggests that POU2F3-positive SCLC may have a different prognosis than its neuroendocrine-positive counterparts. However, the nature of the tumour microenvironment (TME) and its impact on immunotherapy response in POU2F3-positive SCLC (SCLC-P) remain controversial. On one hand, some studies suggest that low-neuroendocrine SCLC (a group that includes SCLC-P) is an immune-’hot’ phenotype with high T-cell infiltration and PD-L1 expression, making it a promising candidate for immunotherapy [31,32]. This view is complicated by several factors. For instance, the immune-active SCLC-I (Inflamed) subtype, which lacks both neuroendocrine and POU2F3 markers, may confound this association [33]. On the other hand, conflicting reports indicate that a more immune-rich TME and better OS are found in SCLC subtypes expressing conventional neuroendocrine markers or low POU2F3 expression [34–36]. Further complicating the picture, a study by Velut et al. concluded that SCLC molecular subtypes do not predict immunotherapy efficacy [37].

Our findings contribute to this complex discussion. In our subgroup analysis, POU2F3 expression was associated with a favourable prognosis in patients treated with traditional chemotherapy alone. However, this prognostic benefit was lost in patients who received chemoimmunotherapy. There are two primary explanations for this discrepancy. First, the smaller number of studies in the chemoimmunotherapy group (*k* = 2 for OS and PFS/RFS), and this issue is compounded by the fact that POU2F3-positive tumours are relatively rare, which further limits the effective sample size and statistical power in a meta-analysis. Alternatively, this finding could imply that the addition of immunotherapy has a less favourable, or potentially even detrimental, effect on POU2F3-positive tumours, thereby negating the prognostic benefit observed with chemotherapy alone. Regardless of the cause, our results challenge the notion that POU2F3-positive SCLC is an inherently immune-responsive subtype that benefits from immunotherapy regimens.

Extrapulmonary NEC is a heterogeneous group of tumours that are morphologically and genetically similar to SCLC. For example, mutation of *TP53* and *RB1* was frequent in various extrapulmonary NECs and SCLCs. However, difference in clinically, genetically, and immunologically features are also found both within extrapulmonary NECs and compared with SCLC. Rb1 loss and p16 overexpression are found more often in genitourinary small cell NECs than in small cell NECs of other organs [38]. In bladder small cell NECs, *TERT* promoter mutation is common but not in other NECs and SCLC [39]. Moreover, Patients with genitourinary small cell NECs had better OS than those with gastrointestinal tract small cell NECs [38]. In SCLC and extrapulmonary NECs, expression level of Phospholipase C Gamma 2(PLCG2), a transmembrane signalling enzyme that produces DAG and IP3, has been strongly associated with POU2F3-positive subsets [8,18,40]. High expression of PLCG2 was associated with increased invasiveness, stem-like features and prometastatic potential in SCLC [40]. This suggests that PLCG2 could be a potential therapeutic target for POU2F3-positive SCLCs. However, PLCG2 or POU2F3 expressions were not associated with metastatic status or response to chemotherapy in bladder NECs. Moreover, POU2F3-positve NECs resembled ‘immune cold’ microenvironment in SCLC, which represents low density of tumour-infiltrating lymphocyte infiltration or PD-L1 expression [41]. This finding contrasts that of cervical small cell NECs, where HPV infection is the primary aetiology, in which POU2F3-positive subtype displays an ‘immune hot’ phenotype with active immune cell infiltration [42]. This discrepancy underscores that the prognostic and immunological characteristics of POU2F3-positive tumours are not universal but are determined by the unique biological context and aetiology of the primary organ. Therefore, it is crucial to avoid uniformly applying the SCLC paradigm when understanding and developing treatment strategies for extrapulmonary NEC, emphasizing the necessity of an organ-specific approach.

Despite our best efforts to provide valuable insights through this systematic review, several limitations must be acknowledged. First, heterogeneity among the included studies poses a significant challenge. Variations in study design, the organs involved, associated tumour types, detection methods for POU2F3 expression – including differences in immunohistochemical protocols, antibodies used and types of biomaterials – and criteria for defining POU2F3 positivity may have introduced bias and affected the comparability of results. Second, studies that did not report dichotomous POU2F3 versus non-POU2F3 expression, such as survival analysis from Qi et al. [22], were excluded. Third, the lack of standardized reporting on treatment modalities across studies may have confounded the analysis, as variations in drug regimens and surgical methods could potentially influence survival outcomes independently of POU2F3 expression. Finally, our study has a significant imbalance in the data between SCLC and extrapulmonary NECs. Our meta-analysis included fourteen studies focused on SCLC but only two study on extrapulmonary NECs. Consequently, while our findings are robust for the SCLC population, they cannot be generalized to all extrapulmonary NECs. The conclusions drawn regarding extrapulmonary NECs are therefore speculative and highlight a critical need for more research focused on this patient group.

## Conclusions

Our study suggests that POU2F3 expression in SCLCs is associated with improved survival, but not when considering both SCLCs and extrapulmonary NECs. Additionally, POU2F3 positivity was associated with younger age and male sex, but not with combined histology or smoking. These findings highlight the distinct characteristics of POU2F3-positive tumours and emphasize the need for standardized detection methods. To clarify the role of POU2F3 as a prognostic marker and therapeutic target, future research should focus on SCLC and, critically, expand to include more studies on the diverse group of extrapulmonary NECs.

## Supplementary Material

Supplemental Material

Supplementary Figure 1.tiff

Supplementary Figure 2.tiff

PRISMA checklist.docx

## Data Availability

The data generated in this study are available upon request from the corresponding author.
