# Supplementary material for: Prognostic values and clinical significance of POU2F3 expression in neuroendocrine carcinomas: a meta-analysis with a focus on small cell lung cancer
Source: Ann Med. 2025 Sep 10;57(1):2556253. doi: 10.1080/07853890.2025.2556253 (PMC12427452; doi:10.1080/07853890.2025.2556253)
Supplement: Supplemental Material [file IANN_A_2556253_SM8452.docx]

Search strategy for PubMed

("POU2F3"[tw] OR "SCLC-P"[tw]) AND ("Carcinoma, Neuroendocrine"[MeSH] OR "carcinoma"[tw] OR "neuroendocrine carcinoma"[tw] OR "NEC"[tw] OR “neuroendocrine neoplasm”[tw])

**Supplementary Figure1**. Subgroup analysis for OS (left plots in A to E) and PFS/RFS (right plots in A to E). (A) Studies using only surgical specimen. (B) SCLC studies that used both surgical and biopsy specimen. (C) Studies that included chemotherapy patients. (D) SCLC studies that included chemoimmunotherapy patients. (E) SCLC studies that used only whole slide to evaluate POU2F3. (F) SCLC studies that used both TMA and whole slide to evaluate POU2F3. (G) Studies that defined SCLC-P subtype in OS. (H) Studies that POU2F3-positive criteria other than SCLC-P in OS. (I) Studies with only extensive stage SCLC in PFS/RFS.

**Supplementary Figure2.** Subgroup analysis for OS (left plots) and PFS/RFS (right plots). (A) Studies that extracted survival data from univariate Cox regression. (B) Studies that estimated survival data from Kaplan-Meier curves.
