# Supplementary figures and images for: Prognostic values and clinical significance of POU2F3 expression in neuroendocrine carcinomas: a meta-analysis with a focus on small cell lung cancer
Source: Ann Med. 2025 Sep 10;57(1):2556253. doi: 10.1080/07853890.2025.2556253 (PMC12427452; doi:10.1080/07853890.2025.2556253)

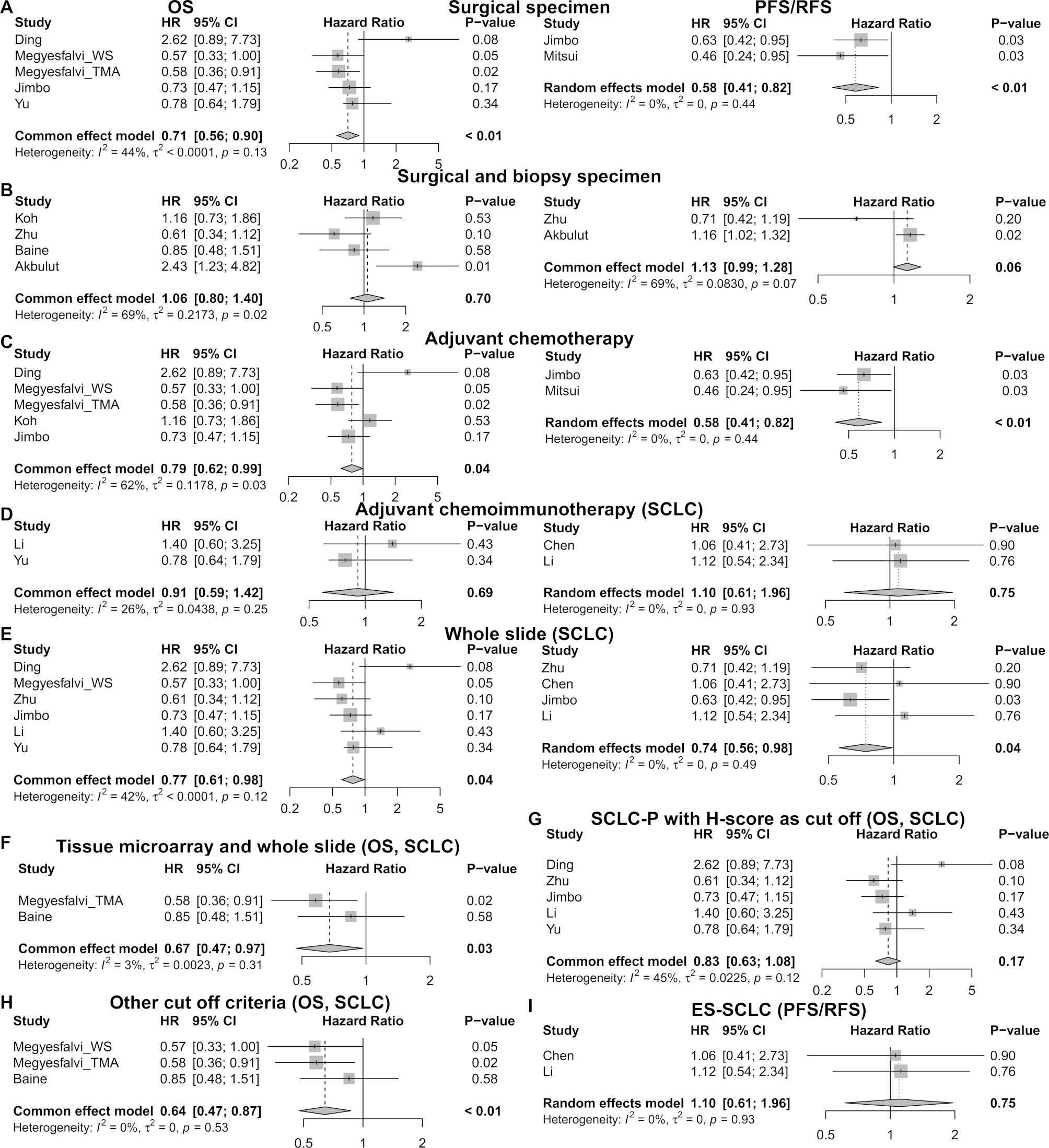

Supplement: Supplementary Figure 1.tiff [file IANN_A_2556253_SM2930.tiff]
